# COVID-19 Vaccination Hesitancy among Healthcare Workers—A Review

**DOI:** 10.3390/vaccines10060948

**Published:** 2022-06-15

**Authors:** Christopher J. Peterson, Benjamin Lee, Kenneth Nugent

**Affiliations:** 1 School of Medicine, Texas Tech University Health Sciences Center, 3601 4th St., Lubbock, TX 79430, USA; ben.lee@ttuhsc.edu; 2 College of Engineering, Texas Tech University, 2500 Broadway, Lubbock, TX 79409, USA; 3 Department of Internal Medicine, Texas Tech University Health Sciences Center, 3601 4th St., Lubbock, TX 79430, USA; kenneth.nugent@ttuhsc.edu

**Keywords:** health care worker, COVID-19, vaccine, hesitancy, mandate

## Abstract

The COVID-19 pandemic and its associated vaccine have highlighted vaccine hesitancy among healthcare workers (HCWs). Vaccine hesitancy among this group existed prior to the pandemic and particularly centered around influenza vaccination. Being a physician, having more advanced education, and previous vaccination habits are frequently associated with vaccine acceptance. The relationship between age and caring for patients on COVID-19 vaccination is unclear, with studies providing opposing results. Reasons for hesitancy include concerns about safety and efficacy, mistrust of government and institutions, waiting for more data, and feeling that personal rights are being infringed upon. Many of these reasons reflect previous attitudes about influenza vaccination as well as political beliefs and views of personal autonomy. Finally, several interventions to encourage vaccination have been studied, including education programs and non-monetary incentives with the most effective studies using a combination of methods.

## 1. Introduction

The COVID-19 pandemic has generated significant interest in vaccine development and effectiveness, as well as public health policies related to the use of vaccines. This discussion has also highlighted the issue of vaccine hesitancy and skepticism. Many individuals choose not to receive the vaccine despite fatalities from COVID-19, assurances of vaccine safety and efficacy, and public health or employer mandates to do so [1]. While vaccine skepticism is not new, the discussion regarding vaccines has recently intensified due to a global pandemic and a new vaccine to address it. Furthermore, while public vaccination skepticism has been well analyzed [2], vaccine hesitancy among healthcare workers (HCWs) has also become apparent.

Healthcare workers typically have several qualities that would presumably predispose them to vaccine acceptance; these include advanced education in the sciences, clinical experience, and membership in professional societies that support vaccination. Furthermore, many medical professionals are at the forefront of the pandemic, both directly observing the effects of SARS-CoV-2 infection and placing themselves at greater risk of exposure. It may, therefore, seem surprising that some medical professionals have chosen not to receive the vaccine, with some even adopting or promoting incredible theories about the pandemic and the associated vaccine. Proponents of HCW vaccination argue that unvaccinated HCWs place themselves and their patients at increased risk of illness [3]. Furthermore, healthcare professionals are viewed by their communities as leaders in the field and thus examples of good healthcare practice as well as trusted sources of vaccine-related information [4]. If HCWs are hesitant about the vaccine, patients are likely also hesitant. Finally, greatly publicized resistance in a small group of highly visible individuals in healthcare professions may also contribute to vaccine hesitancy in the general population.

Given its impact on the public, there have been various approaches to discussing the issue of HCW COVID-19 vaccination. Although logically sound arguments for vaccination are readily available, some commentators have made broad generalizations and even exaggerated the motives and reasoning of vaccination skeptics. Resorting to ad hominem attacks and caricaturing of skeptics by vaccine advocates will likely be unconvincing and only further separate the public from reliable scientific and medical sources. In reality, the reasons for vaccine skepticism are varied and complex, and are often interwoven with personal emotions, identities, and world views. Concerns are unique to every individual and range from concern over side effects and efficacy to fears over governmental abuse of power, to more outlandish theories, such as microchip implantation [5,6]. These issues also intersect with many important philosophical and ethical issues, such as personal autonomy, epistemology, and utilitarianism [7]. Religious and political ideologies are often invoked by those who feel that the moral cost of vaccination outweighs the possible medical benefits. Indeed, beliefs about vaccination are variable such that no single approach or argument is likely to persuade those opposed to it. Participation in vaccination by vaccine-hesitant HCWs will likely occur only after these varied viewpoints are understood and appropriately addressed, thus warranting a thorough investigation into the motivations behind such hesitancy.

This article discusses the causes of COVID-19 vaccine skepticism, traces their development throughout the pandemic, and proposes solutions to promote higher vaccination rates among healthcare professionals.

## 2. Methods

The PubMed (https://pubmed.ncbi.nlm.nih.gov/) and Google Scholar (https://scholar.google.com/) databases were searched from 1 March 2022 to 1 April 2022 for the following terms: “COVID-19” AND “vaccine” AND “hesitancy” OR “attitude” AND “healthcare worker”, “physician”, “nurse”, and “hospital staff”. Additional topic-specific searches were performed as needed. Pre-prints were generally excluded, with some exceptions. Narrative accounts were included from non-academic databases and sources such as news media, blog posts, and official reports.

## 3. Pre-COVID-19 Vaccine Hesitancy among Healthcare Workers

Vaccine hesitancy among HCWs is not new. Many pre-COVID-19 studies have examined HCW vaccine hesitancy, with the majority of this work focusing on seasonal influenza vaccination [8,9]. Before COVID-19, vaccine-hesitant HCWs expressed many concerns similar to those observed during the pandemic, including concerns about safety, mistrust of employers and government authorities, and violation of personal autonomy [8,9]. Intervention programs to encourage HCW vaccination, as well as vaccine mandates, have been previously studied and employed [10].

The 2009 H1N1 influenza pandemic provides as close an approximation as possible to the ongoing COVID-19 crisis, both involving a worldwide pandemic and the rapid development of a vaccine to counter it [11]. Some HCWs were hesitant to receive the H1N1 vaccine over concerns it was developed too quickly [12] and surveys showed that as low as 31% of HCWs were willing to obtain the H1N1 vaccine [13,14]. Though the CDC has recommended influenza vaccination for HCWs since 1981 [15], vaccination rates at first were low in general, in part due to influenza vaccination being largely voluntary. However, recent data indicate much higher vaccination rates (overall: 76%, physicians: 91%, nurses: 90%, assistants/aides 65%) in part due to vaccine mandates [16]. Data have shown that influenza vaccination is effective at preventing illness among HCWs [17] and patients [18,19]. In one study, offering influenza vaccination to HCWs increased vaccine uptake among HCWs (51% with recommendation vs. 5% without), and this difference in HCW vaccination rates was associated with a small though significant decrease in mortality of long-term care residents, despite no difference in non-fatal infection rates. [19]. Additional studies have shown low-quality evidence for a significant effect of HCW influenza vaccination on all-cause mortality as well as “flu-like” symptoms [18,20] and reduced doctor consultation and hospital admissions for “flu-like” symptoms [21]. Skeptics of these results have argued that a computational model has shown HCW vaccination to only have a marginal impact on mortality [22], and others have questioned the quality of this evidence [23]. A rebuttal to these critiques by Hayward points out that death from influenza is hard to track due to the secondary reporting status of influenza. Influenza has the effect of increasing acute illness in older patients with most of the associated mortality being attributed to preexisting conditions rather than influenza. This same paper also points out the relatively weaker effect of vaccines in generating immunity among elderly individuals as an argument for the necessity for HCW vaccination to protect these individuals [24]. Many have drawn comparisons between the influenza and COVID-19; indeed, many of the arguments for vaccination against one highly contagious respiratory virus may be applied to the other.

Vaccination mandates were established in many instances on an institutional level before COVID-19. Vaccinations for healthcare workers against diseases such as hepatitis B, measles, mumps, rubella, pertussis, varicella, and influenza have long been strongly recommended by most public health organizations and regulatory bodies [25,26]. Many institutions and schools, including health profession schools, have historically required HCWs and students to be vaccinated before starting employment or matriculation [27]. In 2005, Poland et al. presented several arguments about mandated influenza vaccination that parallel discussions about COVID-19 vaccination today, including the morbidity and transmissibility of influenza, limitations on scheduling coverage for sick workers, the effectiveness of work requirements in increasing vaccination rates, and the ethical and moral duty of healthcare workers [28]. Despite this duty, one study found that family medicine physicians were more motivated by institutional pressures to vaccinate than the hope of providing protection for their patients [29]. It has also been argued that HCW vaccination requirements establish an example of HCWs as leaders in good preventative medicine practices [25].

Arguments against mandated HCW vaccination include violation of personal liberty [30], questions about the safety and effectiveness of vaccines [31] overestimation of vaccine likelihood to cause adverse effects to those who we rely on for healthcare, and a typical underestimation by HCWs of their own risk of adverse effects from diseases [32,33]. Indeed, several court cases have examined HCW vaccine mandates. For example, H1N1 vaccination requirements for HCW in New York were met with several lawsuits, with plaintiffs arguing that mandates violated, among other things, personal liberty and freedom of religion [34].

Ultimately, workplace vaccine mandates have proven the most successful intervention at increasing influenza vaccination, and this success was enhanced when combined with other interventions, such as education [35]. On the other hand, influenza outbreaks are relatively common, historically occurring in 30–60% of long-term care facilities each year [36] and studies have been inconclusive so far about whether vaccinating HCWs against influenza actually prevented infection [37]. Research has also found that a need for increased quality and quantity of evidence has been a common request from HCWs when confronting decisions regarding vaccinations and whether to recommend them [8]. Indeed, to counter the overwhelming sources of fear and misinformation that confront HCWs, policymakers and administrators should highlight the multiple sources which address the specific questions and concerns of providers and their patients [8]. Quality education and transparent decision-making have also been found to be important preparation for other more compulsory interventions [8,25,32]. Interventions that enhance institutional accountability, such as having a formalized exemption review process, manager-level compliance tracking, and heightened institutional accountability also can increase vaccination, and may also be effective [38]. Ultimately, pre-COVID-19 vaccine hesitancy among HCWs provides important precedence for understanding hesitancy during the current COVID-19 pandemic.

Healthcare workers are often asked to make recommendations for others; for example, 95.9% of Finnish HCWs reported accepting all vaccinations for their children before the pandemic [39]. Among this pronounced majority, however, 13% reported hesitating and 6.3% reported postponing a vaccination for their children. This same group of workers reported that they very rarely guided hesitant patients toward vaccination, and it was found that their recommendation behavior was related more to personal attitudes toward vaccines than trust in other HCWs’ recommendations and competence [39].

Vaccine hesitancy historically may be influenced by both the relative familiarity with and perceived personal risk from diseases. For example, HCWs typically have better uptake of vaccines for diseases less common in the general population, such as measles, tetanus, and hepatitis B [25]. In contrast, the vaccine against the more familiar influenza virus has commonly been involved in HCW hesitancy [40]. A survey of HCW influenza vaccination attitudes and behaviors noted that the main reason HCWs cited for receiving influenza vaccinations was the perception of a risk to themselves and not necessarily consideration of risks to their patients [40,41]. This could simply be because HCWs are not confident the vaccine will protect patients. However, the idea that a disease would not likely pose a personal risk to HCWs may outweigh the potential for protecting vulnerable patients from harm when HCWs make decisions about influenza vaccinations [32,42].

The COVID-19 pandemic provides a rather unique situation involving a new pathogen and high worldwide infection rates with significant medical and economic impact. A review of trends in hesitancy before COVID-19 helps identify which aspects of HCW hesitancy are new and evolving and which have been seen prior.

## 4. The COVID-19 Pandemic and Vaccine Development

**Figure 1:**
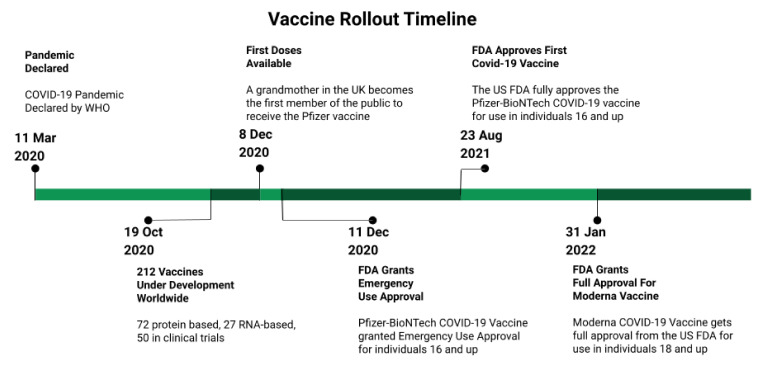
A brief timeline of selected events related to the vaccine rollout.

The COVID-19 pandemic created a rush to develop a vaccine against SARS-CoV-2. The World Health Organization declared a global pandemic in March of 2020 [43]. By late 2020, there were over 200 COVID-19 vaccines in development and over 40 vaccines were undergoing clinical trials [44]. The first vaccines were available for high-risk groups in late 2020. Countries invested billions of US dollars in vaccine research and development, [45] with Phase I trials beginning in April 2020 [46]. The most notable of these vaccines include BNT162b2 (produced by Pfizer-BioNTech) and mRNA-1273 (produced by Moderna, Inc., Cambridge, MA, USA), which use recombinant mRNA technology. Of note was both the speed with which these vaccines were developed and the fact that they were the first approved vaccines using mRNA technology. The first doses of the vaccine were administered to a member of the public on 8 December 2020 [47]. In the United States, these vaccines were granted emergency authorization by the Federal Drug Administration (FDA) on 11 December 2020 (Pfizer) and 18 December 2020 (Moderna), respectively [48]. Due to limited supplies, certain populations, including HCWs, were prioritized to receive the vaccine first. The FDA subsequently granted full approval for both of these vaccines in August of 2021 (Pfizer, New York, NY, USA), and January 2022 (Moderna) [49,50]. Healthcare workers faced significant challenges during the pandemic, including higher workloads, shortages of personal protective equipment, increased risk of COVID-19 illness, burnout, and emotional distress [51]. A COVID-19 vaccine would presumably receive widespread support among healthcare workers as a way to provide personal protection and limit viral transmission, morbidity, and mortality. However, studies before vaccine rollout indicated notable hesitancy among HCWs [52,53,54,55,56], with those willing to vaccinate ranging from 29.7–76.9% prior to availability across a variety of countries, including Belgium, Canada, and Italy (Figure 1).

When vaccines did become available, many healthcare workers were quickly vaccinated. Long-term care facilities were also prioritized, and by 17 January 2021, a median of 37.5% of staff members, and an astonishing 77.8% of residents in long-term care facilities had received at least one dose of the vaccine [57]. Surveillance data from over 2000 US healthcare facilities found at least 50% of workers across facilities were vaccinated by mid-March 2021 [58]. Eventual acceptance varied across the different professions, from vaccination rates as high as 75% among doctors to the lowest rate of vaccine acceptance among a cohort of nurses (56.7%) and nursing aides (45.6%) [59] in March 2021. On the other hand, despite the AMA reporting a 96% vaccination rate among doctors by June 2021 [60] a significant number in the healthcare field chose not to vaccinate, with general rates of vaccination plateauing at 70% in September of 2021, and slowly growing after that to only 77% by December 2021 [61].

**Figure 2:**
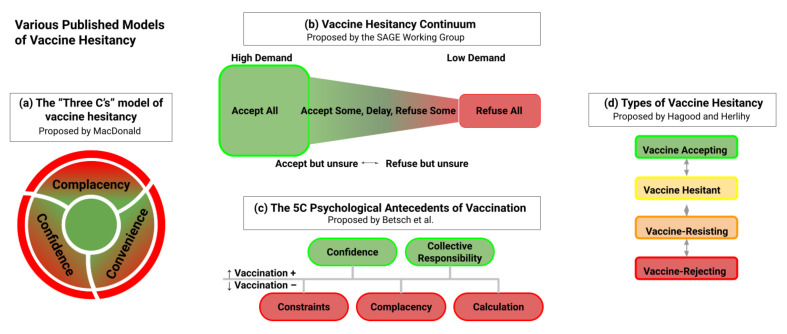
A compilation of the various models proposed to describe vaccine hesitancy. (**a**) The “Three C’s” model of vaccine hesitancy proposed by Macdonald, (**b**) “Vaccine Hesitancy Continuum” proposed by Sage Working Group, (**c**) “The 5C Psychological Antecedents of Vaccination” proposed by Betch et al., and (**d**) “Types of Vaccine Hesitancy” proposed by Hagood and Herlihy.

Ranges of vaccine hesitancy have been described. For example, Hagood and Herlihy suggest three categories—vaccine-resisting, vaccine-hesitant, and vaccine-rejecting [62]. Other classifications have focused on categories related to hesitancy, such as the 3C (complacency, convenience, and confidence) [63] and 5C models (complacency, confidence, constraints, calculation, and collective responsibility) [64], and the Vaccine Hesitancy Matrix (contextual influences, individual and group influences, and vaccine/vaccination specific issues) [65]. While these classifications will not specifically be employed here, they indicate the spectrum of attitudes among those who do not readily support COVID-19 vaccination (Figure 2).

Vaccination hesitancy has persisted despite these vaccines receiving full approval in several countries and hundreds of millions of doses being administered worldwide [66]. As the pandemic has progressed, the discussion has focused on the role of employers and governments in encouraging, if not mandating, vaccination. The discussions around these ideas have interacted with various political and philosophical ideologies and generated discussion, debate, and strong opinions on both sides of the argument [67]. For example, in the United States, attempts to mandate vaccination were met with resistance. In September 2021, the Biden administration announced its intent to mandate COIVD-19 vaccination for HCWs. On 5 November 2021, the Centers for Medicare and Medicaid Services (CMS) also ruled that staff working at Medicare or Medicaid-certified facilities would be required to be fully vaccinated by 4 January 2022, barring an exemption [68]. It was argued that HCW vaccination both protected workers and patients, with data showing reduced infection and transmission in healthcare settings [69]. For example, a large study of Spanish nursing homes found COVID-19 vaccination was associated with an 80–91% reduction in SARS-CoV-2 infections among residents, staff, and HCWs during the 5-month surveillance period [70]. Twenty-four states challenged this ruling with the Supreme Court initially ruling that these states would be exempt from the renewed mandates [71]. On 13 January 2022, the Supreme Court ruled (5–4) that the CMS mandate to be enforced in all states, reversing the interim ruling, with the current deadline for an initial dose being 14 February 2022, with employees fully vaccinated by 15 March [72,73]. Nevertheless, issues around HCW COVID-19 vaccine hesitancy persist and the demographics of these hesitant individuals are varied.

## 5. Demographics of Vaccine Hesitant HCWs

Demographics of HCWs receiving or declining the COVID-19 vaccine, both before and after vaccine distribution, have been widely studied. Positive predictors of COVID-19 vaccination include caring for COVID-19 patients [74] and male sex [74,75,76]. Negative predictors include occupation as a nurse [74] or working at a skilled nursing facility [77,78]. Two studies were observed.

However, some studies have found contradictory results. For example, some found that younger ages [55,79,80,81] were more associated with vaccination; conversely, others observed the same for older ages [54,82,83]. The reasons for this discrepancy are unclear. Older HCWs would be more amenable to vaccination due to the greater risk for severe disease, whereas younger HCWs may be more hesitant due to greater exposure to social media [84] and may be more prone to risk-taking behaviors [85]. On the other hand, younger HCWs may be more receptive due to more liberal political views compared to older adults [29]. One study noted that older HCWs were more likely to vaccinate across all professions except for physicians, but younger physicians were more likely to vaccinate. The authors suggest this may be due to younger physicians, including house staff, being more involved with patient care than older physicians [83]. Interestingly, some studies noted that older individuals had lower perceived COVID-19 vulnerability than younger HCWs [54,75], which is unusual given that older age is a risk factor for COVID-19 morbidity and mortality [86]. Conversely, the presence of underlying conditions also positively affected vaccine acceptance [81] which may be partly responsible for the observation of greater vaccine acceptance among older individuals, who likely have more comorbidities than a younger demographic group.

Education and scope of practice are also associated with hesitancy; HCWs with more education and advanced practice scope (particularly physicians) were more likely to support vaccination [74,75,79,80,81,87,88] though some studies have found no association [89]. More advanced levels of education may result in a greater understanding of COVID-19 research and more resistance to conspiracy theories. However, some authors suggest that accessing reliable information, rather than education, is a better determinant of acceptance and hesitancy [90]. Indeed, knowledge about the COVID-19 illness and vaccine was also associated with vaccination acceptance [85,90]. This could explain why HCWs with higher education are more likely to vaccinate, although information regarding vaccine safety and efficacy is widely distributed and readily available. Rather, this may be due more to increased experience with the analysis of clinical research in some professions.

Race and ethnicity have been widely studied regarding the pandemic. Much like the general population [91]. Black and Latino healthcare workers generally had higher rates of hesitancy than White HCWs [80,92,93,94] with some exceptions. This has been attributed to several factors, including the previous mistreatment of racial and ethnic groups and socioeconomic disparities [91,95]. Interestingly, one study found no ethnic/racial differences between COVID-19 vaccine acceptance among physicians, suggesting that minority physicians might serve as role models to address hesitancy among other HCWs in their racial/ethnic group [83].

It is unclear if exposure to COVID-19 is associated with increased vaccination since some studies have found a positive association between direct patient care (including care of COVID-19 patients) and vaccination [55,82,83,96], while others have found greater hesitancy among those caring for patients [97] or no correlation [88,92,98]. One systematic review found that a majority of included studies (20/35; 57.1%) found that caring for COVID-19 patients or having higher risk perception/fear of COVID-19 was associated with decreased hesitancy [99]. Unsurprisingly, vaccinated HCWs also perceived greater benefits from vaccination, including protection from illness [80]. These observations may be due to differences between medical professions, with some studies finding direct exposure leads to lower rates of vaccine hesitancy in physicians [100] and advanced practice providers [79] but not in other healthcare professions. Studies on HCWs with a previous history of COVID-19 infection are also mixed, with some studies finding HCWs were more [101] or less [75] likely to vaccinate. One study found that HCWs in the emergency department were more likely to be vaccinated compared to other departments [84], possibly due to exposure to patients whose COVID-19 status is unknown. Risk perception is considered an important factor in determining vaccine acceptance. Unsurprisingly, some studies have found the perceived risk of COVID-19 was associated with vaccination [54]. However, some studies have found that risk perception was low in a notable number of HCWs. For example, a survey at an Italian medical center observed that 27% perceived that COVID-19 is not dangerous and 65% believed they were not at risk for infection [55]. Another study of HCWs in Saudi Arabia found that 41.9% of respondents had little or no risk of contracting COVID-19 [102]. One study noted that a majority of unvaccinated participants considered themselves at low risk [103]. Furthermore, exposure to COVID-19 might actually make HCWs more complacent toward COVID-19 and decrease their sense of personal risk, possibly due to having “survivorship bias” from direct exposure to COVID-19 [104].

The clinical setting may also have a role. One study noted higher rates of vaccination among HCWs at hospitals (66%) and outpatient clinics (64%) compared to doctor offices (52%), nursing homes, or care facilities (50%). Home health workers had even a lower rate at 26% [105]. Interestingly, some of the highest rates of vaccine hesitancy were observed in nursing homes or long-term care facility staff [106] which care for elderly individuals who are at higher risk for severe disease. For example, one study found that among long-term care staff, only 44.9% were willing to receive the vaccine as of November 2020, with clinical staff less likely than certain non-clinical staff such as house staff and administration (this study included both clinical and non-clinical staff) [107]. Hesitancy has also been noted among HCWs in the military [108] and at rural hospitals [109]. One study found that the only hospital department or setting associated with higher rates of vaccination worked in the intensive care unit (ICU) [110]. Thus, direct exposure to the severe COVID-19 illness, such as in the ICU, may be a stronger motivator for vaccination.

Higher income [111,112,113] and increased years of work experience [111] are positive predictors. Income may be due to associations with higher educational attainment, leadership positions, advancement in career, and greater perceived benefit from continued employment. It may also reflect healthcare leadership, who may feel more pressure or responsibility to vaccinate due to the visibility of their positions and the precedent their vaccination status sets for those they lead.

Previous vaccination habits, particularly regarding influenza, were correlated with support for/receiving COVID-19 vaccination [54,56,76,114,115]. This suggests that hesitancy for the COVID-19 vaccine may be rooted in previous vaccine hesitancy. Nevertheless, some vaccine-hesitant HCWs contend that COVID-19 vaccine hesitancy is a separate issue, in part because they have previously received other vaccines and chosen to vaccinate their children. This suggests that hesitancy is complicated and cannot always be connected to previous vaccination habits.

Political affiliation is associated with vaccine hesitancy. For example, one study of HCWs in Chicago found a significantly higher rate of vaccination among Democrats than Republicans (63% vs. 19%) [80]. This result mirrors what has been observed in vaccination rates for the general public [116]. This may be for a variety of reasons, although political polarization during the pandemic likely has had a role [117]. This polarization may cause people to reflexively line up along ideological or political party lines rather than assessing decisions individually. Indeed, one author noted that some in the general public may view their vaccine hesitancy as an identity, connecting them with larger groups of individuals who share similar values about vaccination, healthcare, and personal autonomy [118].

Media consumption, particularly social media, has been a major source of speculation and even misinformation regarding the pandemic and vaccination [119]. Some HCWs feel that the media has exaggerated the severity of the pandemic or COVID-19 [110]. A study of Ethiopian healthcare found that social media was more frequently viewed by HCWs as the best source of COVID-19 vaccine information (53.4%) compared to government websites (19.0%) [84]. Some studies have found an association between social media use and COVID-19 vaccine misinformation in the general public [120,121] possibly because these platforms can spread misinformation and isolate users from other sources and arguments. Though misinformation can be spread through these platforms, social media is also used by government and public health institutions, such as the Centers for Disease Control (CDC), to share information about the pandemic. These and other institutions developed campaigns to educate the public about the COVID-19 pandemic and associated public health practices and vaccines. Therefore, it is possible that HCWs are subject to the same social forces that allow for the rapid spread of misinformation through the public at large, and that by virtue of association with certain kinds of people, may end up falling into the same algorithmic echo chambers and disinformation campaigns that promote fringe sources and ideas [122]. This is also interesting, as HCWs presumably have access to more reliable information as “insiders” in the medical community, such as institutional data and subscriptions to academic journals. However, one study found a lack of quality resources as a significant factor in nurses’ hesitation to recommend the HPV vaccine [123]. Therefore, while some HCWs may have access to better information, it is only likely to be useful if these HCWs access, trust, and correctly interpret these sources.

The influence of close social ties has also been examined as a source of hesitancy. Surprisingly, one study found that a history of acquaintances becoming ill, being hospitalized, being admitted to the ICU, or dying from COVID-19 was not associated with vaccine acceptance [85]. Furthermore, if hesitancy was reported in the HCW’s family, friends, or colleagues, the HCW was more likely to also be vaccine-hesitant [85]. It is unclear if this is the result of peer influence on HCW attitudes and vice versa, or if similar attitudes about vaccination tend to cluster together due to social, geographical, or other features. Individuals may also be insulated within groups with similar thinking, thus preventing significant contact with or creating suspicion of outside ideas. This likely occurs to some degree for most individuals, as people tend to interact with those who share similar values and ideas; however, behavioral studies have shown that suspicion and fear decrease, and a more meaningful exchange of ideas between opposing sides can be facilitated by extended contact. Family or household size was also related to vaccine hesitancy, with larger households being associated with higher rates of hesitancy [110]. Interestingly, HCWs with children were more likely to vaccinate themselves [124,125]. This contrasts with studies of the general population, where parents were less likely to vaccinate compared to non-parents [126,127]. Among parents, being an HCW is associated with a willingness to vaccinate children [128,129]. This has been observed among the general public and may be related to socioeconomic factors [130]. Perceptions of whether vaccines primarily are for individual or community protection may also drive behaviors; those who view vaccines as primarily for individual protection may be less likely to vaccinate to support public health measures [131].

Lack of access or difficulty receiving vaccines has been examined, though this is not likely to be an issue for most HCWs [92]. However, HCWs that work several part-time jobs at multiple facilities have reported finding time to vaccinate more difficult [132]. During the early phase of vaccine rollout when distribution was limited, some HCWs who were not employed by a major hospital or healthcare organization may have lived a significant distance from these sites [104]. Financial barriers to receiving the vaccine are unlikely to be an issue in areas where the vaccine is freely available and where employers will allow for HCWs to be vaccinated.

Finally, it should be noted that HCWs are typically more willing to be vaccinated than non-HCWs [133,134,135,136]. While vaccine hesitancy among HCWs has been described as alarmingly high, the number HCWs who had no intention of being vaccinated typically has been in the minority, ranging from approximately 1–5% [82,133,137,138]. This suggests that many HCWs could be persuaded to vaccinate.

## 6. Reasons for Hesitancy

Reasons for hesitancy are varied but grouped across several themes in HCWs, many of which are also by the general public. Several of these major themes are discussed here, but this list is not comprehensive (Table 1).

### 6.1. Concerns about Safety and Efficacy

One of the most frequent reasons for COVID-19 vaccine hesitancy is concerns about safety [80,81,84]. Some of these concerns involve the FDA emergency approval process [79]. These vaccines received emergency FDA approval, a process that has been used during previous public health emergencies [143]. These vaccines later received full FDA approval in August 2021 (Pfizer) [49] and January 2022 (Moderna) [144]. Reports of adverse events, though rare, likely influenced hesitancy. For example, reports of anaphylactic reactions occurred from December 2020–January 2021 [145]. Other highly publicized adverse events included fatal thrombus events in patients receiving the Johnson and Johnson and AstraZeneca vaccine [146] and myocarditis (predominantly in young men) [147]. Fictitious side effects were also circulated, including modification of human DNA, cancer, and infertility [148]. In April 2021, the FDA decided to halt the administration of the Johnson and Johnson vaccine due to six fatal cases of thrombosis in patients who had received the vaccine [149]. It was later resumed on 23 April 2021, after an FDA investigation [150]. In March 2021, several European countries suspended the Oxford–AstraZeneca vaccine also due to concerns about thromboembolic events [151]. Following an investigation by the European Medicines Agency [152]. European countries resumed use of the vaccine as of 18 March 2021 [153] with some countries restricting use to older adults [154]. Nevertheless, these publicized side effects and temporary embargoes may have affected public vaccine acceptance. One study found that in the weeks following the suspension of the Johnson and Johnson vaccine, there was a significant increase in concerns about side effects, but there was no measured increase in vaccine hesitancy [155]. Studies have also observed concerns over fertility. Pregnant or trying to become pregnant HCWs had higher rates of vaccine hesitancy; pregnant women were six times more likely to delay and women trying to become pregnant were three times more likely [156]. Concerns about fertility have also been observed in men [157]. However, a study of HCWs in New York did not find differences in hesitancy among different age groups of women, suggesting that higher rates of female vaccine hesitancy may not be simply explained by concerns over fertility, pregnancy, or breastfeeding [90]. Previous negative experiences with vaccines, whether personally or that of a friend or family member [158]. Finally, it is also possible that some HCWs may also hold beliefs that vaccines are generally unsafe, irrespective of events surrounding the COVID-19 vaccine, perhaps contributed to by the fact that some vaccines in the past have been withheld during pregnancy [159] and that initial safety testing for COVID-19 did not include a pregnant cohort and thus initial messaging from the CDC indicated ambivalence towards whether those pregnant should vaccinate [160].

It also must be recognized that not all HCWs are candidates for the COVID-19 vaccination, though the vaccine is recommended for virtually all individuals except those with a severe allergic reaction to the first dose [161]. Cases of significant allergic reactions are scarce in the literature and would not be considered cases of vaccine hesitancy [162]. The rate of medical exemption use by HCWs is unknown, and this may represent a loophole with potential misuse by employees of medical systems, though this has not been studied.

Health care workers may have personally witnessed severe side effects from previous vaccines [163,164]. Although these instances are likely to be very rare, and cannot explain such widespread hesitancy, they nonetheless highlight the impact that personal experience can have.

Efficacy is another often expressed concern [99]. Some HCWs have expressed concern that the vaccines will be ineffective against COVID-19 or that the immunity will not be long-lasting. This may be due in part to initial uncertainty about the duration of immunity from vaccination [165] or the number needed to treat [166]. This is due, in part, to limitations in the data and was not intended to be a definitive statement on the duration of immunity. Later studies did, in fact, confirm the anticipated waning of immunity, thus requiring a booster shot. Reduced effectiveness against variants such as Delta and Omicron may also have lowered confidence [167,168]. The multidose regimen for some vaccines, as well as the later recommended booster dose, may be seen as an indication of ineffectiveness. This is, however, consistent with previous vaccine schedules, many of which require multiple-dose regimens, such as hepatitis B and polio, as well as seasonal or booster doses, such as influenza and tetanus [169,170]. Breakthrough cases, although typically infrequent and mild [171] may have created doubts about the effectiveness of these vaccines. Finally, results about the effectiveness of COVID-19 vaccines at preventing transmission of the virus have been mixed [172,173,174] possibly due to the effect of variants. These conflicting data and limited effectiveness at preventing transmission from variants may have exacerbated doubt about vaccine effectiveness [103].

### 6.2. Waiting for More Data

Many healthcare workers postponed receiving or deciding about the COVID-19 vaccination until more information about its safety and efficacy became available [139]. This is understandable, as healthcare is a data-driven and evidence-based environment, and a desire to wait for more data before making a decision could be seen as consistent with this. Interestingly, a 2005 study among Dutch parents found that those with higher education and employment in healthcare were more likely to have negative attitudes about vaccination [175]. A 2021 interview-based study among more educated Dutch parents theorized that, rather than being ignorant of scientific principles, may be employing a “critical reflexive” perspective wherein they use scientific methods to question the validity of existing data on vaccination [176]. In other words, these individuals may be applying, in varying degrees, mechanisms of scientific thought such as critical analysis and skepticism to question established results. Using intuition was also a suggested reason for hesitancy among this group [176] highlighting the need to take into account non-data-driven measures, such as emotional responses to vaccination. Healthcare workers may be employing the same reasoning in their decisions to postpone vaccination. However, the initial clinical trials for the Moderna, Pfizer, Johnson and Johnson, and AstraZeneca vaccines included tens of thousands of participants each [177,178,179,180]. This is, in accordance with established standards for FDA clearance, and similar to or exceeds the patient recruitment numbers used in previous vaccine clinical trials [181]. Nevertheless, some may have opted to wait until more information about post-approval adverse events and duration of immunity was available.

It is often unclear what kind of data and how much data would be satisfactory to those postponing vaccination. One sentiment analysis of 3523 responses from HCWs (11% response rate) after emergency use approval, but before widespread vaccine availability found that, among the various sentiments given by the 14.7% of people hesitant to be vaccinated, 29.2% wanted >1 year and 12.2% wanted 1 year of follow-up before feeling comfortable receiving the vaccine; 11.0% said nothing would make them comfortable [182]. Several months after the approval of COVID-19 vaccines in the US, some HCWs still expressed hesitancy about COVID-19 vaccination despite new data, though several trials had already documented the safety and efficacy of these vaccines [183,184,185,186,187]. It may be that some HCWs are waiting for anecdotal data amongst individuals in their social or professional circles regarding vaccinations. Or they may decide to wait for an indeterminate amount of time based on intuition rather than quantitative data. While it is true that more data can produce more accurate or definitive results, undefined endpoints in some of these contingencies mean that these HCWs risk postponing vaccination indefinitely.

### 6.3. Vaccines Developed Too Quickly

Some HCWs expressed concern that the COVID-19 vaccines were developed too quickly [139]. Healthcare workers may or may not be aware of the lengthy developmental timeframe for pharmaceuticals (average 10–15 years) [188], as well as FDA-cleared pharmaceuticals that have received post-approval black box warnings or been removed altogether [189,190]. This recognition may cause some to be skeptical of the shorter timeframe for COVID-19 vaccine development and the emergency FDA approval. Even with now full FDA approval, some may still feel that the vaccines have not been appropriately studied. Indeed, the development of the COVID-19 vaccine was remarkably fast. The time from pathogen recognition to vaccine development, while varied, often takes several years (and sometimes decades) [46]; the COVID-19 vaccines stand alone as the fastest developed vaccine and the first vaccine developed for a pathogen within a year of discovery [46]. However, several factors likely contributed to rapid development, including previous scientific knowledge of coronaviruses (including SARS), rapid global response to the pandemic, funding, public engagement, early investment in upscaled manufacturing, and substantial scientific effort, high rates of COVID-19 disease (thus providing a wider study population), expedited governmental agency review, multiple vaccine candidates, faster production of mRNA and recombinant adenovirus (rAd) vaccine (compared to other technologies), political, and industry collaboration [191]. The mRNA technology has been under development for decades, with mRNA vaccine development extending back to the mid-1990s [191]. Nevertheless, people adopt new ideas and technologies at different rates, described by the diffusion of innovation theory as a range of attitudes from “innovators” and “early adopters” to the “late majority” and “laggards” (see Malcolm Gladwell’s *The Tipping Point*) [192]. This may explain the hesitancy of some to receive the vaccine, despite reassuring evidence, due to its newness. Indeed, some HCWs have expressed greater concern over newer vaccines [193] or vaccine recommendations [194]. Finally, unfamiliarity with the vaccine development process may contribute to hesitancy. For example, a study of over 12,000 US nurses in October 2020 noted high levels of distrust in the vaccine development process, with 40% stating they were “not confident” in the COVID-19 vaccine development process. Regarding information about the process, 60% stated they were “somewhat knowledgeable” and 30% felt they were “not knowledgeable” [195]. Indeed, if the process of COVID-19 vaccine development appears inexplicable, some may be unwilling to accept the results of this process.

### 6.4. Distrust of Employers, Government, and Healthcare System

Some HCWs have expressed distrust of institutions regulating or mandating vaccinations, including their employers, government agencies, professional medical associations, pharmaceutical companies, and the healthcare system in general [53,140]. For example, one study of Canadian HCWs in December 2020 observed a lack of trust in pharmaceutical companies (35%) and experts (27%) [82]. Another noted low confidence in the pharmaceutical industry [81]. There may be several reasons for this. Some HCWs have expressed mistrust of the pharmaceutical industry due to potentially compromising financial motives [193]. Healthcare professionals are likely aware of shortcomings in the healthcare system, both currently and historically [196]. In biomedical and clinical sciences, these include unethical research, such as the highly unethical Tuskegee syphilis study [197]. There is also suspicion that when financial gains are involved, it is a reason for suspecting dishonesty from those producing, distributing, or promoting the vaccine [198,199,200]. This belief may be influenced by the profits from COVID-19 vaccination (Pfizer reported USD 36.7 billion in COVID-19 vaccine sales in 2021 [201]); recommendations for additional booster shots may have only exacerbated this sentiment among some individuals.

A lack of trust in these institutions was observed before the COVID-19 pandemic. For example, a 2015 study of French general practitioners found that while eight of ten respondents trusted the Ministry of Health, 53% also felt that the Ministry of Health was influenced by pharmaceutical companies and 29% preferred their own judgment to official recommendations [202]. The COVID-19 pandemic may have only furthered this mistrust. For example, a 2021 Medscape survey of physicians and nurses noted that 77% of both nurses and doctors said their trust in the CDC has decreased since the start of the pandemic, and 51% of nurses and 48% of doctors reported decreased trust in the FDA [203]. A survey by the National Opinion Research Center (NORC) (commissioned by the American Board of Internal Medicine) found that 30% of physicians reported decreased trust in the healthcare system and 43% reported a decrease in government health agencies, though 53% stated their trust had remained the same. This decrease in trust was greatest among physicians who already had low trust in the healthcare system [204]. A 2021 Spherix Global Insights report noted decreased confidence in the FDA, with nearly half of physicians reporting decreased confidence in the past year. This may have been related to non-COVID-19 reasons [205] such as recent FDA approval of drugs like Roxadustat, Tenapanor, and Aducanumab [206]. A survey of HCWs in Europe noted that some HCWs felt pressured by the pharmaceutical industry to administer vaccines and felt that representatives were not transparent about side effects [200]. Some of this mistrust may stem from an awareness of previous issues with vaccine safety, including the Cutter laboratory incident (where contamination with live poliovirus led to 40,000 cases of polio, resulting in over 180 cases of paralysis and 10 deaths) [207] as well as the rotavirus vaccine Rotashield (withdrawn due to increased incidence of intussusception) [208].

One question is, why do HCWs continue to work in a system that they mistrust or have low trust in? Some may view themselves as a force that keeps these institutions “in check” by, for example, protesting or refusing to comply with vaccination mandates. Some HCWs hold beliefs or practices outside of established guidelines and evidence. For example, some HCWs believed alternative medicine or homeopathy were effective against COVID-19, even preferable to the vaccine [81]. Preference for alternative medicine over vaccination has been noted for other vaccines as well [209]. Healthcare workers who favor or practice alternative medicine may be an indication of mistrust in the healthcare system and biomedical sciences. It may also reflect of lack of understanding of the evidence for alternative medicine practices or the clinical research process. Finally, HCWs may view their involvement in the healthcare system as a necessary but unfortunate reality. They may be willing to navigate issues they are finding troubling until those issues involve their own healthcare or personal autonomy.

Treatment of HCWs by the healthcare system itself may also contribute to trust (or lack of it). Dr. David Grabowski, a professor of healthcare policy at the Harvard Medical School, suggests that HCW mistrust may be due to a lack of institutional support during the pandemic, such as PPE and hazard pay [132]. Indeed, if HCWs perceive that the healthcare system has failed to support them during a time of crisis, then they may be less willing to comply with additional requirements from these institutions. Frustration over what is perceived as a mishandling of the public health response to the pandemic [210] or concerns over commercial profiteering [211] may have made some HCWs less willing to trust later public health recommendations regarding vaccination. National issues may contribute to this. For example, one study of French HCWs found that some HCWs in French Guiana and the French West Indies had a greater distrust of mainland France, possibly due to the historical treatment of these regions [212]. Still, some HCWs who are skeptical of governments and other institutions may have received other vaccines, possibly due to direct personal experience with these vaccines rather than due to government approval [104].

Of note, a study in September 2020 with HCWs found that scientific evidence was more convincing than expert opinion regarding the COIVD-19 vaccine. Indeed, 86.3% reported that scientific evidence on vaccine safety and efficacy would be the most convincing [133]. Healthcare workers were also more likely to trust the information on COVID-19 vaccines from other HCWs, especially their primary physicians, as opposed to information from hospital management, the federal government, or political authorities [213]. If this is generally true among HCWs, then this may help explain that initially published trials regarding vaccine safety and efficacy were not more convincing.

### 6.5. Infringement on Personal Rights

Some HCWs have expressed concern that COVID-19 vaccination requirements represent an invasion of personal autonomy [214]. Arguments for mandatory vaccination include patient and HCW safety as well as limiting HCW shortages due to COVID-19 illness [3,215]. However, some HCWs feel that the vaccine is a matter of personal choice [79,216] and thus may feel that vaccine mandates are both a government overreach and a violation of their personal rights. For example, a former UCLA anesthesiologist filmed himself being escorted off the UCLA campus for being at the workplace while unvaccinated. He felt that vaccination mandates were a violation of his personal liberty. At a recent anti-vaccine rally, he had encouraged others neither to vaccinate nor apply for an exemption, claiming that to do so would acquiesce to what he considered government overreach [217]. Indeed, other HCWs have expressed concern that vaccine mandates infringe on their personal freedoms. Some have expressed willingness to lose employment over their stance [218]. In June 2021, Methodist Hospital in Houston, TX, terminated 158 employees for failure to vaccinate [215]. Other hospitals have either had employees terminated or resign over failure to vaccinate [219]. For some of these HCWs, issues around vaccination (and more specifically vaccine mandates) may be a moral issue rather than one of personal safety or health, and value patient autonomy over being role models [8].

Some HCWs may perceive the COVID-19 vaccine as separate and distinct from other vaccines, and thus may support certain vaccines while rejecting COVID-19. For example, some COVID-19 vaccine-hesitant HCWs reported receiving all childhood vaccinations, annual influenza shots, and vaccinating their children [220,221]. They may view the issue surrounding the COVID-19 vaccine as a unique and isolated issue, possibly to the novelty of the mRNA technology and rapidity of development. These HCWs may resent the label “anti-vaccine” or “anti-vax”, likely due to its negative cultural and political connotations but also because they have been compliant with previous vaccines and do not want to be associated with those who outright deny the effectiveness of vaccination. One worker describes two conflicting viewpoints, simultaneously attributing a pneumonia vaccine to saving her life from a respiratory illness but refusing to receive the COVID-19 vaccine. This suggests that COVID-19 vaccination is seen by some as a separate entity from other vaccines, and indeed research has shown that hesitancy is not a stable trait and exhibits some level of lability [222]. This is in contrast to studies showing a positive association between previous influenza vaccination acceptance and willingness to receive the COVID-19 vaccine [56,223]. This may be because HCWs classify the seasonal influenza vaccine as less necessary than childhood or adolescent vaccinations, creating thus another category by which vaccines can be perceived. Indeed, one study found that HCWs with specific COVID-19 vaccine hesitancy (generally accepting of vaccines) typically had high confidence in vaccines and exhibited low complacency and a high level of collective responsibility [104].

Some HCWs may be more resistant to vaccine mandates than the vaccine itself and may refuse to vaccinate out of protest. Mandates conflict with libertarian ideals of self-governance and can generate opposition in those with these political leanings [224]. However, given that mandates were instituted after the vaccine had been available for several months, one wonders why HCWs opposed only to mandates would still be unvaccinated by the time these mandates went into effect.

Finally, healthcare workers also have differing opinions on COVID-19 vaccine mandates. While these mandates have been supported by multiple professional organizations in the United States, including the American College of Physicians, American Medical Association, American Nurses Association, and American Pharmacists Association, some remain hesitant [225]. Though institutional support can certainly lend credibility to vaccine mandates, it is unlikely to be effective in those who already have low trust in institutions. In fact, many HCWs are against mandatory vaccination, with surveys showing opposition as high as 57% [55]. However, other HCWs have supported mandates, for reasons including reducing the risk of illness for themselves and patients, especially vulnerable populations [141].

### 6.6. Vaccination Unnecessary

Some healthcare workers have expressed the idea that vaccination is not necessary [226]. Some contend that natural immunity is preferable to immunization [82,198]. Others contend that vaccination should not be required or is not necessary for those who have already contracted COVID-19 [110,142]. This may be due to feeling protected from COVID-19 due to previous illness or because those who are more likely to contract COVID-19 had fewer health-protective behaviors in the first place [157]. One study of over 65,000 US HCWs noted a lower vaccination rate among those who had previously been infected with COVID-19 (OR, 0.55; 95% CI, 0.51–0.58, *p* < 0.0001) [227]. Still, others claim that they do not feel at risk for COVID-19 [102] or that COVID-19 is less serious than depicted [142]. Furthermore, continued public health measures, such as masking requirements, make unvaccinated HCWs skeptical of the vaccine’s effectiveness or the personal utility of vaccinating given some personal protective requirements remained the same [142].

Risk perception may be altered depending on how the issue of vaccination is framed. For example, one study examined Israeli HCWs’ willingness to receive the avian influenza (H7N9) vaccine from different perspectives—if they were a Chinese citizen (where the H7N9 strain emerged) and as an Israeli HCW should the virus spread to their own country. Healthcare workers were more willing to be vaccinated when considering the perspective of a Chinese citizen. The authors suggest that the former scenario distances the HCW from their concerns about vaccinations and makes them more likely to vaccinate. Furthermore, they suggest that “optimistic bias”, or the belief that one is at less risk than one’s peers, may also be influential [228]. Comparisons from this study to a global pandemic have limitations, although it may explain why HCW may support vaccination for others but chose not to be vaccinated themselves. When HCWs do choose to vaccinate, some studies find they may vaccinate to protect themselves rather than patients [229] though HCWs have expressed patient safety as a motivating factor as well [230].

Some may have also incorrectly categorized the vaccine as “experimental” and may feel that their receipt of the vaccine post-approval is participation in an ongoing experiment [231,232] even though monitoring of adverse events continues after FDA approval (known as Phase IV) is a standard procedure for all pharmaceuticals. A small number of HCWs also subscribed to fringe or conspiracy theories about COVID-19, including that COVID-19 is a hoax [110].

It should be noted that medical training does not necessarily lead to the appropriate application of critical thinking or evidence-based reasoning. Indeed, some HCWs may still use a more intuitive or unsystematic approach to medical decision-making [193] one that was more common before the current emphasis on rigorous evidence-based medicine practices was implemented. While it may seem odd that HCW would fail to follow recommendations from public health and professional organizations, as well as their own recommendations to patients, in reality, doctors failing to follow their own advice is not new. For example, a study of HCWs in Jerusalem found that 72.1% of those surveyed supported health clinic staff influenza vaccination, but only 30.2% were themselves immunized [233]. A systematic analysis found that between 2000 and 2014, 21% of HCWs worldwide were tobacco users [234].

### 6.7. Other

Several reasons for vaccine hesitancy are less frequent. One HCW reported feeling bullied by other HCWs and administrative staff for her resistance to COVID-19 vaccination; she eventually started a campaign to protest mandatory vaccination, partly in response to this perceived mistreatment [163]. Some unvaccinated HCWs feel that they are being unfairly criticized and even demonized for not vaccinating, despite having struggled through the pandemic the same as vaccinated workers. One HCW reported feeling disheartened by people who once praised their efforts now treating them as a “dangerous person” because of their vaccination status [218]. Another HCW felt selfish about getting the vaccine before family members were able to do so, saying she would be vaccinated once the vaccine was more widely available [142]. Some feel that vaccination programs are being overemphasized and at the expense of other effective public health measures [235]. Others are morally opposed to vaccine mandates [236] and may cite religious reasons for being vaccine-hesitant [237]. One HCW felt overwhelmed by the amount of information regarding COVID-19 [103]. Indeed, information overload regarding COVID-19 has been studied in the general public and may contribute to difficulty in decision making [238].

Greater rates of vaccine hesitancy have been observed in low and lower-middle-income countries [239] possibly due to limited resources or mistrust of other nations where the vaccine was produced.

## 7. Towards the Future

Interventions for addressing vaccine hesitancy among the general public have been varied and met with mixed results. A systematic review of these interventions found that the most successful interventions are multi-faceted and dialogue-based, though few studies actually quantified the impact of the interventions on vaccination [240]. For example, an intervention by Nyahn et al. uses information provided by the CDC as well as images and narratives of children affected by vaccine-preventable diseases did not affect parental vaccine hesitancy, with images and narratives of sick children increased belief in serious vaccine side effects in those believing in a link between vaccination and autism [241]. Another systematic review found that interventions tailored to specific populations and concerns, including multicomponent and dialogue-based interventions, were the most effective [240]. Dubov et al. identified four clusters of vaccine-hesitant HCWs, including misinformed, uninformed, undecided, and unconcerned, and proposed interventions tailored to each [110]. For example, personal narratives may be more effective for those in the “misinformed” cluster, whereas motivational interviewing may be better suited to those in the “undecided” cluster. Selecting the appropriate intervention to address the specific concern will likely be the most effective approach.

Several interventions for HCWs and COVID-19 vaccines have been studied. An intervention at a medical center in Japan involved multi-faceted approaches including an educational lecture and leaflet distribution regarding COVID-19 vaccination and arranging tests for polyethylene glycol allergy. The center noted an increase in vaccination rate among those who were initially unsure about vaccination (88.9%) and “unwilling” (67.3%). Respondents to the post-intervention questionnaire felt that the information leaflets (88.2%) and e-learning opportunities (84.2%) were the most effective [242]. A review of interventions at over 400 US nursing homes found that nursing homes with higher coverage provide non-monetary rewards, paid time-off, goal setting, and expert-led educational sessions. Facilities that employed more strategies had higher vaccination rates, and facilities that employed several strategies had higher vaccination rates [243].

Non-monetary incentives (such as t-shirts or granting freedoms) have also been effective [243,244]. Monetary incentives have had mixed results in the general population [245,246,247], and they can be complicated by ethical concerns [248]. Other recommendations include harnessing social media, acknowledging concerns, expressing empathy, and avoiding using dogmatic, moralistic tones when discussing vaccination [249]. Sending out reminders or email “nudges” as one institution did, might also be useful [250]. Organizations have also reported success in increasing vaccination rates using “drives” or institution-wide vaccination campaigns [115].

Several government and public health organizations have provided recommendations for addressing HCW COVID-19 vaccine hesitancy. The Ontario COVID-19 Science Advisory Table listed several recommendations on how to promote vaccination among HCWs, including explaining the vaccine development process, normalizing feelings of anxiety, identifying HCWs who have chosen to vaccinate, and easy access to vaccination [251]. The CDC recommended encouraging vaccination among senior leaders, hosting discussions where HCWs can ask questions about vaccination, and providing small, non-monetary rewards for vaccination [252]. Doing so may foster an environment of greater transparency and cooperation. Leaders who set an example of vaccination have been shown effective for influenza vaccination [253]. This may help build trust among those who have a suspicion that administrators are acting out of self-interest or being disingenuous. Indeed, in conversations with vaccine-hesitant HCWs, it is important to remember the challenges HCWs have made during the pandemic and their contribution to healthcare. It is unlikely that dismissing or ignoring these contributions due to a lack of vaccination will be effective. Furthermore, creating an environment where HCWs are free to express their concerns is important. Some HCWs may avoid voicing their concerns due to fear of inviting ridicule from colleagues or institutional pressure or punishment [254].

Willingness to vaccinate has also increased over time [58,81,139] suggesting that additional HCWs may vaccinate with time. One study noted that 59.7% of HCWs who had been unsure about vaccination were later vaccinated by a 6-month follow-up [255]. However, it is unclear in what timeframe this will occur [255]. Furthermore, the benefits of allowing HCWs to delay vaccination or continue to seek exemptions must be weighed with the continued risks of being unvaccinated. This discussion may become less relevant if cases decline before a widespread change in HCW hesitancy occurs. If future COVID-19 vaccinations will be needed (like the annual influenza shot), prolonged use of these vaccines may increase confidence.

It is important to remember that most HCWs are typically not trained as experts in immunology and vaccinology. Although they may have substantial clinical experience administering vaccines, this does not necessarily translate to understanding the principles behind vaccine development. Lack of exposure to the rigors, complexity, and decision-making involved in clinical trials and public health measures may also impact hesitancy and confidence in vaccine safety and efficacy research. For example, a study of vaccine hesitancy among HCWs in Uganda suggested that in countries where vaccine and other clinical trials may be limited, HCWs may have low trust in the clinical research process [256]. One study of HCWs in Ethiopia from May 2021 noted that 39.5% of participants believed that they could become infected with COVID-19 from the vaccines [257].

Healthcare workers who received or planned to receive the COVID-19 vaccine were positively influenced by people around them, including colleagues, supervisors, personal providers, and close associates [80]. For example, the group Nurses Who Vaccinate, an organization that advocates for evidence-based vaccine information, was formed in 2011 by a nurse Melody Butler who was hesitant to receive the influenza vaccine during the 2009 H1N1 pandemic. It was not until a nurse educator addressed her questions that she decided to vaccinate; she hopes that nurses modeling acceptance of vaccination and constructive dialogue will have a similar effect [258]. Indeed, dialogue-based interventions have been effective in addressing vaccine hesitancy. This may also influence HCWs who rely more heavily on interpersonal or anecdotal experiences regarding vaccination.

Vaccine mandates are effective at increasing vaccination rates, both for COVID-19 [259,260] and influenza vaccines [10,261] though studies on the impact of COVID-19 vaccine mandates are limited at this time. Among HCWs, one study of radiology departments with a site-specific mandate noted an improved vaccination rate as the institutional deadline approached [262]. However, effective mandates do not address ethical concerns and must be balanced with the recognition that a minority of HCWs may quit rather than comply. Mandates may increase mistrust and resentment toward the healthcare system even among those who do vaccinate. Historically, vaccine mandates have been perceived negatively by some HCWs [263]. In response to this, some mandates for influenza vaccination have given employees the option between vaccination and an alternative, such as wearing surgical masks, avoiding patient contact, or avoiding high-risk patients [10,264]. One study from 2009 found that HCW compliance with influenza vaccination increased with mandates to wear masks when working with infected individuals [265]. However, some HCWs may actually prefer to wear a mask [209], meaning this strategy may not be feasible in situations where mask-wearing is required regardless of vaccination status or if complete vaccination coverage is the goal. Nevertheless, sites with no vaccine requirement, on-site vaccination, or on-site promotion had significantly lower rates (42.1%) for the 2018- 2019 influenza season than the total vaccination rate among all centers [266]. Despite the effectiveness of mandates, disruptions in care have occurred due to HCW shortages from termination due to non-vaccination. For example, in September 2021, the Lewis County hospital system in New York paused maternity services due to staffing shortages from mass resignations due to the vaccine mandate [267]. This is contrasted with shortages due to HCW illness [268]. Although mandates may be necessary to increase institutional vaccination rates, there will always be those who will still resist vaccination.

Pressure to vaccinate may only exacerbate vaccine-hesitant attitudes. One study of HCWs and social care workers in the UK found that pressure to vaccinate actually increased hesitancy [103]. Another study noted that vaccine mandates were associated with higher levels of reactance, or negative emotions regarding a perceived loss of freedom, and lower rates of COVID-19 vaccine intention [269].

Finally, although HCWs are often seen as distinct from the general public, they are susceptible to the same emotions, biases, concerns, and assumptions that the general population has. A survey of HCWs in Turkey in January 2021 noted some respondents expressing feelings of anxiety, hopelessness, and helplessness regarding the pandemic and issues around vaccination [232]. Stresses from the pandemic, both professionally and personally, may lead to burnout and possibly higher rates of complacency regarding health-protective measures. While medical training and experiences can lend a unique view of disease, it does not necessarily remove these other influences and concerns. It is also possible that some individuals will not receive the vaccine under any circumstances.

## 8. Conclusions

This review has several limitations. The changing and ever-evolving landscape of the pandemic may result in previous observations being less relevant as the pandemic progresses. Furthermore, surveys of HCWs performed at different times in the pandemic may not be comparable, as these studies are from different countries, healthcare systems, and different cohorts of professionals. However, consistency across many studies regarding reasons for hesitancy suggests that these differences may not be extensive. Finally, support for vaccination does not always translate into action. For example, a follow-up of a survey of HCW influenza vaccination intent found that six-fold fewer HCWs were vaccinated than intended [133]. Finally, though there are many studies regarding interventions amongst vaccine-hesitant members of the general population, few studies exist for HCWs. While some recommendations have been proposed, more data are needed to determine which interventions are most effective in this unique demographic.

In conclusion, the reasons for COVID-19 vaccination hesitancy among HCWs are complex and varied. These concerns are consistent with those observed for other vaccines and in the general population, but the rapid development of the COVID-19 vaccines using novel technologies created unique elements for hesitancy during the pandemic. When addressing vaccine hesitancy, it is important to recognize the variety of concerns an HCW may have. Furthermore, medical training and clinical experience do not remove HCWs from the same emotions and dilemmas that all members of the general population experience. Thus, despite their professional titles, concerns about vaccination should be acknowledged while simultaneously holding professionals to high standards. Achieving this balance is difficult and will likely involve trial and error. Nevertheless, organizations and individuals interested in promoting vaccine acceptance should be aware of the complexity of vaccine hesitancy and the effect of various interventions. Discussions about HCW vaccination are likely to continue, both regarding COVID-19 and other vaccines. Future studies should continue to examine effective interventions for not only increasing vaccination rates but changing fundamental attitudes leading to vaccine hesitancy.

## Figures and Tables

**Table 1:** Representative surveys analyzing vaccine hesitancy.

Author	Survey Date	Country	Participants Number	Response	Author’s Conclusion
Verger [53]	October–November 2020	France, Belgium, Canada	2678 (Physicians and Nurses)	48.6%—high acceptance 23.0%—moderate acceptance 28.4%—hesitancy or reluctance Main concern- safety	Must build trust about efficacy and safety
Biswas [99]	February 2020–January 2021 35 different studies	Worldwide	HCW 76,741	22.51% hesitant Range 4.3–72.0% Main concerns: side effects, safety, efficacy	Education and policy-based interventions are needed to ensure vaccination
Meyer [139]	December 2020	United States	HCW 16,292	55.3% will receive 16.3% will not 28.4 % unsure Intentions to receive increased after EUA recommendation	Highly visible information from experts may increase intent
Pal [140]	February–March 2021	United States	HCW 1374	7.9% hesitant Mistrust important factor 83.6% would accept an annual booster	Concerns about safety and efficacy and lack of trust underlie hesitancy
Bell [103]	January 2021	United Kingdom	HCW SCW 1917	6.6% declined vaccine offer Complex analysis of characteristics of participants	Authors offer detailed policy recommendations
Woolf [141]	April–June 2021	United Kingdom	HCW 5633 total 3235 answered free text question	18% favored mandatory vaccination	Building trust with education and support may be effective with hesitant HCW
Janssen [81]	December 2020–March 2021	France	4349 HCWs	Online survey presenting hypothetical scenarios for efficacy, longevity, and adverse events. Quantified the effect of each on willingness.	Fear of adverse events was main concern, hesitancy decreased with time. Reassurance about adverse events is important.
Choi [142]	March–May 2021	United States	2948 HCWs surveyed, with semi-structured interviews	Nurses less likely than physicians to see vaccine as safe or effective. Many claiming vaccines unnecessary or unsafe.	Stressed education and mandates

HCW—health care workers, SCW—social care workers, EUA—emergency use authorization.

## Data Availability

Not applicable.
